# Current Approaches for Genetic Manipulation of *Streptomyces* spp.—Key Bacteria for Biotechnology and Environment

**DOI:** 10.3390/biotech14010003

**Published:** 2025-01-02

**Authors:** Sergii Krysenko

**Affiliations:** Valent BioSciences, Biorational Research Center, 1910 Innovation Way, Suite 100, Libertyville, IL 60048, USA; sergii.krysenko@valentbiosciences.com; Tel.: +1-(224)-563-7863

**Keywords:** actinobacterial genetic engineering, *Streptomyces*, biotechnology, primary metabolism, secondary metabolism, synthetic biology

## Abstract

Organisms from the genus *Streptomyces* feature actinobacteria with complex developmental cycles and a great ability to produce a variety of natural products. These soil bacteria produce more than 2/3 of antibiotics used in medicine, and a large variety of bioactive compounds for industrial, medical and agricultural use. Although *Streptomyces* spp. have been studied for decades, the engineering of these bacteria remains challenging, and the available genetic tools are rather limited. Furthermore, most biosynthetic gene clusters in these bacteria are silent and require strategies to activate them and exploit their production potential. In order to explore, understand and manipulate the capabilities of *Streptomyces* spp. as a key bacterial for biotechnology, synthetic biology strategies emerged as a valuable component of *Streptomyces* research. Recent advancements in strategies for genetic manipulation of *Streptomyces* involving proposals of a large variety of synthetic components for the genetic toolbox, as well as new approaches for genome mining, assembly of genetic constructs and their delivery into the cell, allowed facilitation of the turnaround time of strain engineering and efficient production of new natural products at an industrial scale, but still have strain- and design-dependent limitations. A new perspective offered recently by technical advances in DNA sequencing, analysis and editing proposed strategies to overcome strain- and construct-specific difficulties in the engineering of *Streptomyces*. In this review, challenges and recent developments of approaches for *Streptomyces* engineering are discussed, an overview of novel synthetic biology strategies is provided and examples of successful application of new technologies in molecular genetic engineering of *Streptomyces* are highlighted.

## 1. Introduction

### 1.1. Microbial Diversity as a Basis for Metabolic Diversity

Bacteria form the basis of the biosphere and many physiological processes (e.g., chemolithotrophic growth, specific fermentations, nitrogen fixation, methane formation, anoxygenic photosynthesis, secondary metabolite production of antibiotics or toxins, etc.) are limited to prokaryotes. Bacteria possess a much larger genetic potential than any other group of organisms on Earth [1]. For example, the genome of the pathogenic actinobacterium *Mycobacterium tuberculosis* features 376 unique proteins out of a total of 3995 putative ones (approx. 9.4%) [2], which do not show similarities to any known proteins in other organisms. In combination with the above-mentioned advantages, bacteria, especially representatives from the phylum Actinobacterium, became an important source in the search for new biocatalysts and natural products. The search for desired metabolic performance usually first leads to the natural habitat of the organism involved. The occurrence of certain nutrients that supply nitrogen, carbon, phosphate, sulfur and other precursors for primary and secondary metabolism represents ecological niches for organisms that have specialized in the utilization of these molecules during evolution. It determines nutrient supply for cells in the respective ecological niche and, therefore, colonization by certain species of microorganisms that can utilize these nutrients [3,4].

Microbial community formation is influenced by environmental conditions, physiological stress and the interaction with various species [5,6]. With 10^4^ to 10^7^ bacteria/g, organisms from the genus *Streptomyces*, phylum Actinobacteria, are one of the main representatives in soil [7]. Actinomycetes have adapted to a large variety of ecological niches, including terrestrial, intracellular, aquatic, artificial and others [3,4]. Many species, especially from the genus *Streptomyces*, are capable of polymer decomposition of dead plants, and fungal and animal material [7]. This biological function contributes to the recycling of biomaterials, and the global carbon, nitrogen and phosphate cycle, as well as soil organic matter formation [4,7]. Furthermore, representatives of Actinomycetes (e.g., from the genus *Frankia*) have nitrogen-fixing functions in symbiotic associations with plants or other organisms like insects [8].

### 1.2. Environment of Streptomyces, Biological Natural Product Producers

The natural habitat of species from the genus *Streptomyces* is soil. These bacteria can also form symbiotic interactions with other organisms [9]. One example of a well-studied symbiosis is between *Streptomyces hygroscopicus* and *Aspergillus nidulans*, where the streptomycete serves as an inducer of secondary metabolite production (aromatic polyketides) by the fungus [6,9]. Another known symbiosis between various *Streptomyces* spp. and higher organisms include such with the leaf-cutting ant *Acromyrmex echinatior* [10] and with the solitary digger wasp of the genus *Philanthus* [11].

Furthermore, complex interactions between *Streptomyces* spp. and other organisms take place in the rhizosphere [12,13,14]. Such interactions can be synergistic or antagonistic. In the synergistic interaction, *Streptomyces* spp. may stimulate mycorrhiza formation [15]. Both fungus and bacteria can work equally positively on the plant by providing water and nutrient uptake. In return, the fungus and streptomycete receive carbohydrates from the plant and/or from hyphal exudates. Antagonistic interaction is provided by either the streptomycete or the mycorrhizal fungus for better plant growth, but both can inhibit each other’s development. *Streptomyces* has been reported to be a keystone shaping the bacterial community structure at the surface of arbuscular mycorrhizal fungal hyphae [16].

*Streptomyces* spp. can influence other interaction partners in the rhizosphere by producing antibiotics, for example, combating plant pathogens or breaking down harmful substances [17]. Streptomycetes can secrete the plant growth hormone auxin, having a direct effect on plant growth [9,18]. *Streptomyces* sp. AcM29 inhibits gram-positive bacteria, producing the siderophores ferulic acid and desferrioxamine B and showing a negative influence on the diverse mycorrhizal fungi, while plant pathogenic fungi are not inhibited. *Streptomyces* sp. GB4-2 promotes the growth of the plant-pathogenic fungus *Heterobasidion abietinum*, but at the same time reduces the plant’s defense mechanisms. Furthermore, produced by the *Streptomyces* sp. GB4-2 strain, piceamycin demonstrates inhibitory activity against gram-positive bacteria [19].

In addition, a small number of streptomycetes are known to cause damage to potatoes, sweet potatoes, carrots, radishes, tomatoes and eggplants [20,21]. An example of such a pathogen is *Streptomyces scabies*, which target potato tubers and can cause lesions on the tuber surface due to the production of the phytotoxin thaxtomin [21].

The interaction of *Streptomyces* with other bacteria, like interactions with fungi, also leads to the induction or increase in secondary metabolite biosynthesis. A predatory bacterium that lives on *Streptomyces*—*Myxococcus xanthus*—can induce actinorhodin production during aerial mycelium development in *Streptomyces coelicolor*. Such inductions could also be caused by other bacteria, such as *Bacillus subtilis* [22]. Other bacteria, such as *Amycolatopsis* spp. AA4 or *Streptomyces venezuelae*, can influence the prodigiosin or siderophore biosynthesis in *Streptomyces coelicolor* [22].

### 1.3. Primary Metabolism in Streptomyces as an Important Part of Secondary Metabolism

To cope with variable nutrient conditions in their natural habitat in soil, *Streptomyces* can utilize a remarkably large variety of C-sources, such as monosaccharides, polyols, disaccharides, amino acids, sugar alcohols, deoxy sugars, glycosides, dicarboxylic acids, ketoacids [23,24,25,26] and amines like ethanolamine and polyamines [27], as well as N-sources, such as ammonium, nitrate, urea, amino acids and amino sugars, as well as monoamines and polyamines [25,26,27]. The mechanism of carbon catabolite repression allows cells to regulate the levels of carbon sources in cells, repress protein synthesis under the presence of a catabolite generated from an exogenous carbon source and protect themselves against wasting the protein-synthesizing machinery [28,29,30]. The availability of carbon and nitrogen sources greatly impacts the morphological development and production of secondary metabolites in *Streptomyces* [25,26,27,28,29,30].

Nitrogen is incorporated into primary and secondary metabolism by key enzymes of nitrogen assimilation: the glutamine synthetases (GSs) and glutamine-2-oxoglutarate-aminotransferase (GOGAT). GSs are active under conditions of nitrogen deficiency, having a high substrate affinity and catalyzing the condensation of ammonium and glutamate, synthesizing glutamine under ATP consumption [27,29]. GOGAT catalyzes the conversion of glutamine to glutamate from glutamine and 2-oxyglutarate. In addition to glutamine synthetases GSI (GlnA) and GSII (GlnII), *Streptomyces* spp. possess a set of GS-like proteins GlnA2, GlnA3 and GlnA4 that play a key role in the metabolism of amines [27]. At high ammonium concentrations, glutamate dehydrogenase (GDH) catalyzes the conversion of ammonium and α-ketoglutarate to glutamate (reductive amination), with the reverse reaction having low substrate affinity [29]. Nitrogen metabolism in *Streptomyces* is strictly regulated at the transcriptional and post-translational levels, being controlled by transcriptional regulators GlnR, GlnRII, AmtR, NnaR, Crp and AsfR [27,31,32,33,34,35], as well as by the signaling proteins PII and GlnE at the post-translational level [33,34,35,36,37].

Another essential nutrient element for metabolism in Actinobacteria, triggering the production of secondary metabolites, is phosphate [38]. In *Streptomyces* spp., the phosphate can be transported by the PstSCAB system, which is mediated by binding phosphorylated PhoP to the promoter region of the PstSCAB operon [38,39].

In *Streptomyces* spp., the gene clusters *pitH1-pstSCAB*-*ppk* are linked to phosphate metabolism, whereas the *ppk* gene was demonstrated to play a negative role in the control of antibiotic biosynthesis in *Streptomyces* [40]. Phosphate control in S*treptomyces* is mediated by the two-component system (TCS) PhoR-PhoP, which orthologs have been found in most Actinobacteria [38,39]. PhoP belongs to the OmpR family of DNA-binding response regulators PhoR-PhoP, controlling primary metabolism and secondary metabolism, which has been reported in *S. coelicolor*, *S. lividans*, *S. tsukubaensis*, *S. natalensis*, *S. avermitilis* and other streptomycetes [38,39,40].

**Figure 1:**
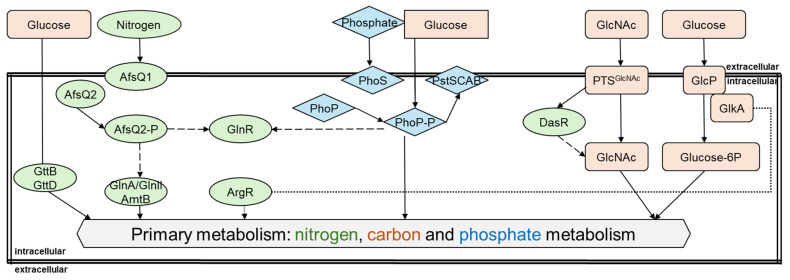
A scheme of the main interconnections of primary metabolic pathways in *Streptomyces*. Arrows indicate positive effects; dashed arrows indicate negative effects. The dotted lines indicate an uncharacterized or indirect regulation. Rectangles and orange color represent parts of the carbon metabolism; ovals and green color—nitrogen metabolism; diamond shape and blue color—phosphate metabolism. Glucose as the main carbon source in *Streptomyces* influences all three main components of the primary metabolism.

Sulfur is another essential element for bacterial metabolism [41]. In *Streptomyces* spp., biosynthesis of cysteine and methionine via homocysteine (Hcy) is linked through transsulfuration pathways, whereas cysteine is the key intermediate in most pathways of sulfur metabolism. Inter-conversion between homocysteine and methionine occurs in the activated methyl cycle (AMC), which involves ATP and additional cofactors cobalamin and 5-methyl-tetrahydrofolate. In *Streptomyces* spp., a direct sulfhydrylation pathway involving *metX* and *metY* has been found (Figure 1) [42].

### 1.4. Streptomyces as Producers of Secondary Metabolites

Actinomycetes have gained attention mainly because of their biosynthetic potential to produce diverse medically and industrially relevant secondary metabolites. With the beginning of the “Golden Age” of antibiotic discovery in the 1940s–1960s, a lot of valuable actinomycetes-derived compounds were discovered, being afterward in many cases developed into commercial agrochemical and pharmaceutical products [43].

*Streptomyces* are capable of producing a wide variety of secondary metabolites, which are not primarily essential for survival, but enable them to protect themselves against certain environmental influences. These include diverse compounds relevant for agriculture, medicine and industry, such as saccharides (e.g., aminoglycosides, lincosamides), nucleologists (aminonucleosides, peptidyl nucleosides), polyketides (e.g., tetracyclines, macrolides), non-ribosomal synthesized peptides (e.g., actinomycin), polypeptides, glycosides, triterpenoids (hopanoids), lipoproteins, alkaloids, polyethers and lantibiotics [44]. Despite the great diversity, many natural products rely on similar biosynthesis principles to be led back. Basic units or preliminary stages are developed through a systematic chemical, regulated production process to form a biologically active molecule. These underlying biosynthetic principles can be found in many different production strains [43,44,45].

Examples of secondary metabolites produced by *Streptomyces* include siderophores, which serve to improve the absorption of iron from the environment, pigments (e.g., melanin, carotenoids), antibiotics (e.g., chloramphenicol, lincomycin, neomycin, streptomycin, tetracycline, albaflavenone), immunosuppressants (FK-506, FK-520), cytostatics, antimycotics/fungicides (e.g., nystatin, natamycin, amphotericin B), herbicides (phosphinothricin), anti-tumor substances (doxorubicin), anthelmintic substances (avermectin), growth promoters in ruminant animal feed (monensin), as anticholesterol and coccidiostatic substances (e.g., lasalocid) and as insecticides (milbemycin), and osmoprotectants (e.g., ectoin) [43,44,45,46]. Specifically, antibiotics feature different modes of action, having an impact on cell wall biosynthesis, membrane transport, RNA synthesis, DNA replication, translation, nucleotide and fatty acid build-up, etc. About 2/3 of all known antibiotics are synthesized by *Streptomyces* [20], but the resources are far from exhausted [46]. The search for new natural substances involves a wide variety of paths taken in addition to the classic, complex biological and chemical screening methods; the numerous genetic and bioinformatics-based methods are preferred nowadays. Genomic approaches are used to search, identify, and investigate secondary metabolite clusters in isolated strains, revealing their full production potential [47]. Further efforts are required to identify the biosynthetic gene clusters (BGCs) encoded in the genome responsible for the biosynthesis of newly produced secondary metabolites. The targeted activation of biosynthetic gene clusters is the preferred approach for the discovery of novel secondary metabolites, which involves engineering cluster-situated regulators, refactoring the target clusters and heterologous expression. Efficient genetic manipulation tools are required for targeted activation of biosynthetic gene clusters [46,47].

Advancements in whole-genome sequencing revealed that the genomes encode many BGCs to produce secondary metabolites. At the same time, only a few of the BGCs are expressed under standard conditions and the biosynthetic potential of a majority of actinomycetes remains unexploited. To address this challenge, methods of natural product discovery, such as isolation and strain cultivation methods, metabolomics and (meta)genomics, as well as new molecular tools for the genetic engineering of Actinobacteria have been introduced [48].

### 1.5. Hosts for Heterologous Expression

*Streptomyces* have been repeatedly reported to be successfully used as hosts for the heterologous expression of BGCs of interest. However, during cultivation and genetic handling, challenges can occur arising from the complex metabolism and genome of *Streptomyces*. Another challenge is the long generation times of typical *Streptomyces*. In both liquid culture and solid media, it takes several days until cell growth has reached the stationary phase. In addition, *Streptomyces* are difficult to transform due to one or more efficient restriction/modification systems [49]. Also, after successful transformation, efficient expression often fails because the host’s own strongly active proteases and synthesized heterologous proteins may immediately degrade. However, for some strains, such as *Streptomyces venezuelae* and *Streptomyces albus*, high-throughput screening and high-frequency transformation protocols could be established, along with optimized handling under laboratory conditions [50].

For improvement in the yields of secondary metabolites, genetically modified *Streptomyces* hosts (also referred to as “super-hosts”) were generated. In such strains, endogenous BGCs and nonessential genes or genomic regions are removed, which results in strains that can conserve energy and precursor building blocks having specific precursor pools. Examples include engineered strains of *S. coelicolor*, *S. lividans*, *S. albus*, *S. avermitilis*, *S. chattanoogensis* [51], *S. tsukubaensis* [52], *S. venezuelae* [53] and multiple others that have shown improved target secondary metabolite production.

Primary metabolism in *Streptomyces* and its importance for the supply of precursors for secondary metabolism was highlighted in other reviews [25,26,27,28,29,30,31,32,33,34,35,36,37,38,39,40,41,42]. Secondary metabolism in *Streptomyces* has received a lot of attention being highlighted in numerous reviews [21,25,43,44,45,46,47,54,55,56,57,58,59,60,61,62,63,64]. New methods for the search for useful compounds produced by *Streptomyces* spp. and recent advancements in approaches for genetic engineering of streptomycetes were covered in numerous reviews during recent years [50,56,60,61,65,66,67,68,69,70,71,72,73,74,75]. Investigations of *Streptomyces* metabolism, genomes and new tools of molecular genetic engineering provided new insights into possibilities to overcome many common challenges of actinobacterial engineering that were considered not solved until recently. This review provides the reader with a comprehensive overview of recent advancements in possibilities to genetically engineer *Streptomyces* with a focus on the establishment of such novel approaches.

## 2. Genetic Tools for *Streptomyces*

Genetic manipulation of *Streptomyces* is crucial for diverse academic and industrial applications. The commonly used strategies of genetic engineering in *Streptomyces* include expression of multiple copies of the whole BGC, refactoring of BGCs by substitution or modification of native regulatory elements, expression of BGCs in optimized native or heterologous hosts, expression of regulatory genes and deletion of genes encoding repressors. Such engineering approaches require genetic manipulation, which depends on available molecular toolboxes for assembling genetic constructs, as well as procedures for their introduction into the host. In the past decades, several bottlenecks were identified that retarded the process of natural product discovery from *Streptomyces* [20,43,50].

Lack of compatible molecular tools, limited cloning and DNA transfer methods, genetic instability, DNA degradation, high GC-content and different codon usage of the newly introduced foreign DNA are obstacles that often prevent the genetic manipulation of actinobacterial strains [50]. Classical approaches include DNA overexpression, deletion, disruption and replacement, as well as the employment of diverse plasmids (e.g., suicide vectors). These approaches require the selection and screening of single- and double-crossover recombination events, making these strategies time-consuming and of low efficiency, since double-crossover mutants are rarely obtained.

Advances in sequencing techniques, approaches for molecular engineering and new bioinformatics tools facilitated the discovery and identification of new BGCs, as well as other promising targets for genetic manipulation in *Streptomyces*. Enzymes for molecular biology applications, cloning strategies, approaches of synthetic biology, methods for transfer of the generated constructs into the cell, new vectors and genetic parts have been recently optimized for application in diverse actinobacterial strains [76,77].

**Figure 2:**
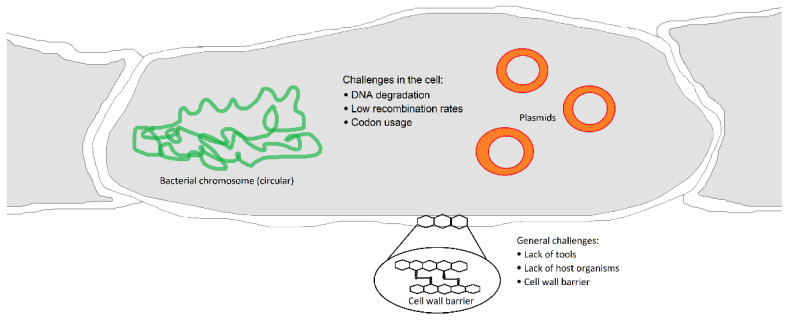
Schematic representation of an actinobacterial cell with potential barriers to genetic manipulation. Many difficulties in *Streptomyces* engineering are connected to general challenges, such as lack of tools for DNA delivery, lack of suitable host strains and complex and multilayered cell-wall barrier. However, many challenges appear in the cell after the DNA transfer, such as degradation of introduced DNA, low recombination rates between the host chromosome and mobile genetic elements (plasmids), as well as different codon usage among strains.

However, key challenges remain in the development of genetic methods for actinomycetes, such as the lack of suitable vectors for DNA transfer, difficulties in the introduction of DNA into the cell by passing the thick cell-wall barrier, restriction of the exogenous introduced DNA, inefficient gene expression due to high GC-content and so on (Figure 2) [56].

To challenge these limitations, different genome editing technologies have been developed: (a) techniques to optimize expression of BGCs in the heterologous host with the acquisition of the target gene cluster from the native host genome (e.g., using a genomic library of cosmids, fosmids, BAC/PAC); (b) techniques for ligation and assembly of BGCs to the vector by sticky/blunt end ligation, Gibson cloning, etc.; (c) techniques to transfer the BGC-encoded vector to the heterologous host for expression by conjugation or protoplast transformation; and (d) techniques to target secondary metabolite production by expression of BGCs of interest in integrative or replicative vectors. Furthermore, during the last couple of decades, novel techniques have been proposed, such as PCR-targeting [78], Cre-loxP recombination system [79], I-SceI promoted recombination system [80] and CRISPR/Cas-based approaches [60]. Discoveries on the clustered regularly interspaced short palindromic repeat (CRISPR)/CRISPR-associated protein (Cas)-based tools have further improved the genetic toolbox of *Streptomyces*. Furthermore, application of genetics parts, such as synthetic promoters (e.g., constitutive *ermE*, *SF14*, *kasO*, *gapdh*, *rpsL* promoters; inducible *tipA nitA*, *xylA* promoters), ribosome-binding sites (AAAGGAGG; native or synthetic RBSs), terminators (e.g., Fd, TD1) and reporter genes (*luxAB*, *amy*, *xylE*, *gusA*; eGFP, sfGFP, mRFP, mCherry) expanded the toolbox for *Streptomyces* engineering (Table 1) [49,50,56,61].

### 2.1. Methods for Heterologous DNA Transfer into Filamentous Actinobacteria

**Figure 3:**
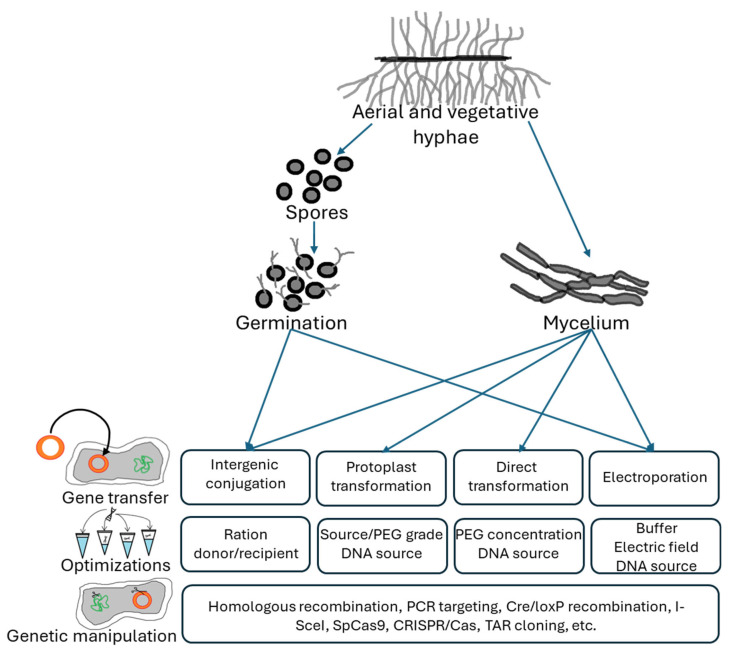
Overview of the DNA transfer systems in *Streptomyces*, including the parameters to be evaluated when applied to genetically manipulating strains. Due to the complex actinobacterial developmental cycle, difficulties in DNA transfer and stability may appear at different stages depending on the target (germinating spores or developed mycelium). Optimization steps that may be undertaken depend on the selected method.

The process of DNA transfer into a cell involves its uptake across the complex cell wall. To overcome this barrier, multiple methods for DNA delivery into actinobacterial cells have been developed, including protoplast transformation, electroporation and conjugation. Protoplast transformation requires the preparation of competent cells as unicellular protoplasts or as mycelium. Electroporation is based on the use of a high-voltage pulse to the suspension of mycelium to induce the formation of membrane pores. Conjugation allows the DNA transfer by cell–cell contact (Figure 3) [20,50,56].

Thus, DNA delivery methods are required to be specifically adapted to a particular strain. The DNA transfer protocols developed for Actinobacteria implemented different strategies to penetrate this barrier. Another obstacle is restriction-modification systems that can interfere with the introduction of exogenous plasmid DNA, e.g., site-specifc endodeoxyribonucleases, restriction enzymes and nuclease-dependent systems. For example, the neomycin producer *S. fradiae* possesses multiple restriction systems. Non-type II restriction and oxidative cleavage were reported as additional challenges to introducing foreign DNA [81]. In troubleshooting efforts, DNA delivery methods have been widely optimized for non-model actinomycetes [20,56].

### 2.2. Synthetic Parts

#### 2.2.1. Integrative and Replicative Plasmid Expression Systems for Actinomycetes—Cloning Vectors for the Genetic Manipulation of Filamentous Actinomycetes

Genetic manipulation of actinomycetes heavily depends on the availability of suitable vectors, such as plasmids, fosmids and cosmids, as well as bacterial or P1-derived artificial chromosomes (BACs, PACs). Such genetic elements are required to support the molecular cloning of DNA fragments, introduction of DNA into the host cells, stable integration in the host cell and expression of the cloned genes. Such mobile genetic elements can either autonomously replicate in the cell cytoplasm or integrate into defined sites of the host chromosome. Although many naturally occurring mobile genetic elements, like bacteriophages, plasmids and transposons, have been found in Actinobacteria, only a few of them were applied to the genetic manipulation of actinomycetes [56,82].

The commonly used vectors can be grouped into two distinct types. First are autonomic replicative vectors featuring different copy numbers and including linear plasmids, as well as suicidal (temperature-sensitive) vectors (Table 1) [65,83,84,85]. The second type is integrative vectors that can carry out the integration of DNA into the host genome via site-specific recombination. Such vectors are either derived from actinophages (e.g., FBT1, FC31, VWB) or actinobacterial integrative/conjugative elements (e.g., AICE) (Table 1) [86,87,88,89,90]. Various resistance markers have been introduced for applications on plasmids for *Streptomyces* engineering. Some of these markers were sources from existing biosynthetic gene clusters. The commonly used marker genes provide resistance against thiostrepton (*tsr*), chloramphenicol (*cat*), hygromycin (*hph*), erythromycin (*mls*) and viomycin (*vio*). Resistance genes originating from transposons of Gram-negative bacteria can also be applied for actinobacterial engineering, such as neomycin/kanamycin (*aphII*) and phleomycin (*ble*) from Tn5, gentamicin (*aacC1*) and apramycin (*aac(3)IV*) [20,91]. These resistance genes are expressed in *Streptomyces* from their own transposon promoters and seem to be functional in a wide range of actinobacterial strains [56,82,92].

*Streptomyces* naturally have a wide variety of conjugative plasmids; however, only exceptionally large plasmids encode biosynthetic pathways for antibiotics. There are “low copy” plasmids and small “multi-copy” plasmids, as well as integrative and conjugative elements of actinomycetes (AICE) [92]. Low-copy plasmids, small “multi-copy” plasmids and AICEs can facilitate the transfer of chromosomal markers (chromosome mobilization ability) with a probability of 0.1–1% [20,75,92].

*Streptomyces* feature high GC content, which makes cloning their DNA difficult. In addition, there are also complex purification steps involved in removing all impurities from the DNA samples isolated from soil (e.g., humic acids), which may lead to an additional drop in cloning efficiency [93,94]. Thus, cloning vectors that have a positive selection are advantageous. This ensures that, during cloning steps, the transformation of respective host organisms works properly. Classical positive selection is based on the SacB system from *Bacillus subtilis* [95] and the RpsL system [96]. However, the SacB system is not suitable for use in *Streptomyces* hosts since it is not lethal for transformants with insert-free vectors, so they cannot be distinguished from transformants that carry vectors with an insert [95]. On the contrary, the RpsL system in *Streptomyces* is functional, but its major disadvantage lies in the laborious and time-consuming mutagenesis that is required for every host organism to introduce streptomycin resistance [96].

An alternative to these systems is the use of inversely repetitive sequences that flank the multiple cloning sites of the vector. In the initial state (“empty” vector), these complementary regions of the plasmid are separated by a region of DNA that acts as a spacer. Subsequently, the spacer can be removed and replaced by a desired DNA fragment. In case of failure of insertion by re-ligation, the free DNA ends do not have a spacer between the inversely repetitive sequence areas anymore. This can happen due to their complementary interaction with each other, e.g., forming a hairpin structure. This hinders replication of the vector in the cell due to the occurrence of the hairpin structure termination loop, which forms a hairpin-like structure acting as a signal to terminate the replication [20,75]. It is of importance for practical use, as all successful transformants should carry a plasmid with a cloned insert. An example of such a classical system is the plasmid pIJ699 [97].

Furthermore, the use of strong promoters in expression vectors is necessary to ensure sufficiently high expression rates. Although an inducible promoter can offer additional advantages, it must be considered that such regulated promoters usually have the problem of lower activity, while strong promoters are often unregulated. This has great genetic potential, especially in soil or humus habitats, where most degradation processes of organic and inorganic material take place, which requires corresponding enzymatic performance [98]. Soil also provides the habitat of most active Actinomycetes, including *Streptomyces*, which possess many enzymes involved in biochemical reactions for the degradation of organic and inorganic material. For example, the *S. avermitilis* genome possesses genes encoding 148 hydrolases, 59 esterases, 10 lipases, 128 proteases and peptidases and 78 oxygenases annotated [99]. Comparable figures apply also to the *S. coelicolor* genome [24].

Inverse repetitive sequences have already been reported to be used as a possibility for positive selection, for example, in the plasmids pIJ699, pJGSF14 and pJGSF15 [20,97]. These inversely repetitive sequences are separated by a spacer, which is replaced by the insert during cloning. The vector without the spacer or insert forms directly adjacent complementary secondary structures that may negatively impact the replication of the vector in the transformed host. A decisive advantage of this principle is its universal application in every bacterial host [92].

pUWL218, pUWL219, pUWL-SK and pUWL-KS shuttle vectors for *E. coli*, and *Streptomyces* are used as expression vectors (Table 1) [100]. Most integrative vectors used in the genetic engineering of Actinobacteria involve phage-derived site-specific DNA recombination systems, such as ΦC31, ΦBT1, VWB, TG1, SV1 and R4 (Table 1) [84,85,86,87,88,89,90,91,92,93,101,102,103,104,105,106,107,108,109,110]. Some are derived from the integrative plasmid pSAM2 (λ-integrase) [92].

The ΦC31-derived vectors are able to integrate into the host genome at the *attB* site, but also when the correct *attB* site is missing [101]. At lower frequencies, such integration occurs at multiple locations via pseudo-attachment sites. Thus, the ΦC31-derived integrative plasmids might have a broader host range. To have the possibility to use two compatible integrating vectors in the same organism, other similar vectors have been constructed based on the integrase genes and *attP* sites of other actinophages, such as ΦBT1, SV1 and VWB [54,56,92,101].

Vectors harboring the ΦBT1 site can integrate at the unique *attB* site, which is localized in the *sco4848* gene of the *S. coelicolor* genome or its orthologues in other *Streptomyces* spp. [102]. Integration of a plasmid at this position into the *Streptomyces* genomes inactivates *sco4849* and the co-transcribed gene *sco4848.* The most common integrative vectors used in *Streptomyces* are derived from the *S. coelicolor* A3(2) phage ΦC31, such as pSET152, pIJ8600, pOJ436 and pOJ444. Furthermore, alternative integration sites were proposed, e.g., from the actinophage ΦJoe, which encodes a serine integrase and belongs to the largest *Streptomyces* phage R4-like clusters. Applicability of the ΦJoe system was demonstrated in in vitro recombination assays, as well as in vivo in *S. venezuelae* and *E. coli* (Table 1) [56,60,76,92,103].

Recently, several vectors were introduced based on the concept of modular construct assembly or “plug-and-play” strategy. For instance, SEVA (Standard European Vector Architecture) was introduced as a web-based resource developed for easier construction of plasmids based on a fixed architecture. The modular concept of SEVA, as well as the public availability of sequences, for exchanging the modules within different vectors is advantageous for the high-throughput generation of plasmids for the engineering of *Streptomyces* [111].

#### 2.2.2. Promoters

In *Streptomyces*, constitutive promoters are extensively used for research and industrial applications (e.g., expression of BGCs), since they generate constant gene expression levels regardless of the growth phase. The most used strong promoter is ermE*, which is a derivative of the *ermE* promoter and contains a trinucleotide deletion in the ermEp1 region of the erythromycin resistance gene from *S. erythraeus* [95]. Other used promoters include KasO from the SARP family regulator in *S. coelicolor* A3 and SF14 from the *S. ghanaensis* phage I19 genome, as well as promoters *gapdh* and *rpsL* from *S. griseus*, which have higher activity than the ermE* promoter (Table 1) [81,82,106,112,113].

To develop strong constitutive promoters, screening strong synthetic promoters from a randomized promoter library has been shown to be an effective approach. The synthetic *kasO* promoter was designed based on a library that was used to generate synthetic promoters with promoter strength of 0.95–187.5%, compared to the native parental *kasO* promoter [114]. Also, on the *ermE* and *actII*-*orf4* promoters of *S. coelicolor*, such an approach has been conducted, but synthetic promoters were still weaker than the ermE* promoter [115]. Strong promoters can also be identified using gene expression data; for example, in *S. albus* and in *S. coelicolor*, promoter sequences of genes with strong expression were selected based on their transcriptional profile, resulting in the selection of promoters that were stronger than the ermE* promoter [116].

Furthermore, controllable gene expression systems were established in *Streptomyces*. The most widely used inducible promoter in *Streptomyces* is the *tipA* promoter induced by thiostrepton. The expression level of the *tipA* promoter is considerable. This limits the precise regulation of the targeted gene expression, but its expression is sometimes used to maintain low expression levels of toxic genes [117]. Another example is the tetracycline-inducible promoter tcp830, which was constructed by combining the *ermE* promoter and Tn10 *tetR*/*tetO* systems [118]. Other inducible promoters include the PA3-rolO and P21-cmt promoters. The PA3-rolO promoter is resorcinol-inducible and is a combination of the *rolO* operator and synthetic promoter PA3. The P21-cmt promoter is a cumate-inducible system synthesized by fusing the operator of the *Pseudomonas putida* F1 cumate degradation operon to the P21 synthetic promoter [119]. Further inducible systems in *Streptomyces* include *nitA* [120] and *xylA* [121] promoters (Table 1). The *nitA* promoter originating from the nitrilase promoter of *Rhodococcus rhodochrous*, can be induced by ε-caprolactam and the transcription regulator NitR. The *xylA* promoter is regulated by xylose [120].

#### 2.2.3. Reporter Genes

Characterization of genetic parts in *Streptomyces* requires reporter systems, which allow for rapidly representing gene expression levels with almost no influence on cell physiology. Many antibiotic resistance genes have been used as conventional markers for gene expression. However, the effects of such genes on cellular metabolism and specific dynamic range are pitfalls when using these systems. For the quantification of gene expression, colorimetric methods are preferable since gene expression levels can be quantified. Various colorimetric methods have been developed for *Streptomyces*, e.g., based on *luxAB*, *amy*, *xylE* and *gusA* (Table 1) [50,51,61,122,123,124].

*gusA* is the most widely used colorimetric reporter system in *Streptomyces*, but colorimetric reporter systems based on an enzymatic reaction require an additional substrate treatment, which may negatively affect cellular metabolism [51]. For instance, catechol dioxygenase (encoded by *xylE*) produces hydroxymuconic semialdehyde from catechol. On the other hand, fluorescent proteins do not require supplemental reagents and are suitable for screening in *Streptomyces*, for example, GFP derivatives, such as eGFP and sfGFP, and mRFP, mTagBFP, mCerulean, mTFP, mCherry, mKate and mCardinal (Table 1) [125,126,127,128]. Since *Streptomyces* have relatively high levels of background autofluorescence, several studies have argued that fluorescent proteins are not suitable for high-throughput screening of *Streptomyces* [61,128].

#### 2.2.4. Ribosome-Binding Sites

Protein levels in cells do not directly correlate with mRNA abundance and depend on translational efficiency. Thus, transcriptional regulation alone is considered to be insufficient to design an efficient gene expression system in *Streptomyces* [129]. The development of a translational regulatory genetic part is in the initial stage. Transcriptional and translational genetic parts with various strengths must be combined to be utilized to design and control enzyme expression in gene clusters. Translational efficiency is primarily determined by the 5′ untranslated regions (5′-UTR), ribosomal binding site (RBS) and codon usage of target genes. RBS contains the Shine–Dalgarno (SD) sequence, which includes a complementary sequence with the 3′ end of the 16S rRNA region of the 30S ribosomal subunit. Accessibility and diversity of the SD sequence influence binding affinity with the ribosome and determine translational efficiency (Table 1) [130].

An example of an RBS search and design is a study conducted in *S. venezuelae*, in which sequences of the strongest RBS, among 15 native RBSs, were selected and randomized. Subsequently, 177 synthetic RBSs with activities over 200-fold were compared to their parental RBSs. Seven promoters were combined with nine RBSs in a pairwise manner for screening of the most optimal promoter-RBS set for gene expression. Another example is a selection of two promoters and four 5′-UTR sequences that was conducted in *S. coelicolor* based on TSS-seq, RNA-seq and Ribo-seq. Pairwise sets of promoters and 5′-UTR sequences demonstrated strength in a range of 0.03–2.4-fold, in comparison to the ermE* promoter containing the SD sequence of the *nitA* gene [131].

#### 2.2.5. Terminators

Only a limited number of terminator sequences are available for *Streptomyces*. One example is TD1 from *Bacillus subtilis* phage Φ29. Other examples include Fd, which is a bidirectional transcription terminator originating from *Escherichia coli* phage fd. Lambda T0 and T7 terminators have been used in multiple *Streptomyces* vectors, but such terminators have not been systematically validated in *Streptomyces* for effects on gene expression levels. Transcription terminators play crucial roles in recycling transcription complexes and in gene expression levels. Recently, Term-seq, which is an RNA sequencing method enabling genome-wide determination of transcript 3′ end positions, was proposed as a suitable technique for screening terminator sequences in *Streptomyces* genomes (Table 1) [50,61,132,133,134].

#### 2.2.6. Riboswitches for Biosensors

A possibility to regulate gene expression is to use natural or modified riboswitches. Riboswitches are non-protein coding RNAs. They can regulate diverse cellular processes, including transcription and translation. These regulatory parts at the 5′-region of mRNA control the gene expression based on allosteric alterations of the structure. Riboswitches are specific to different ligands. This feature was leveraged for the development of biosensors as a synthetic biology tool (Table 1) [135].

Biosensors are composed of a signal input module (transcription factors or riboswitches), a regulatory module like transcription factor-dependent promoters and a signal output module, such as reporter genes [136,137]. Application of the theophylline-dependent riboswitches was reported for *S. coelicolor* [138]. Furthermore, an antibiotic-specific whole-cell TetR repressor-based biosensor has been introduced for screening and optimization of some antibiotic producer strains, such as pamamycin producers [50].

**Table 1:** A combined list of synthetic parts for genetic manipulation of *Streptomyces*.

Name	Category	References
pRM5, pUC119, pOJ446, pKC1139, pHU204, pIJ101, pIJ699, pJGSF14, pJGSF15, pUWL218, pUWL219, pUWL-SK, pUWL-KS	Replicative plasmids	[65,83,84,85,100]
pSET152, pMS82, pOJ436, pJ10257, pIJ6902, pSBAC, pIJ8600, pOJ444, pIJ10702, pHL931, pCAP01, pESAC13, pCLY10	Integrative plasmids	[86,87,88,89,90]
ΦC31, ΦBT1, Bxb1, ΦOZJ, VWB, SV1, RP3, Φ1/6, R4, Φjoe, TG1	Integrases	[101,102,103,104,105,106,107,108,109,110]
*ermEp*, ermEp*, *SF14*, *kasO*, kasOp*, *gapdhp*, *rpsL*, *thlM4p*	Constitutive promoters	[61,81,112,113,114,115,116]
*tipA*, *tcp830p*, *otrBp*, *nitAp*, *P21-cmt-CymR*, *xylAp*, *PA3-rolO-RolR*	Inducible promoters	[117,118,119,120,121]
*xylE*, eGFP, sfGFP, mCherry, *luxAB*, *gusA*, mRFP, mTagBFP mCerulean, mKate, mCardinal	Reporter genes	[51,122,123,124,125,126,127,128]
AAAGGAGG	Ribosome-binding sites	[129,130,131]
TD1, Fd, ttsbiB	Terminators	[61,132,133,134]
Synthetic theophylline-dependent riboswitch	Riboswitches/Biosensors	[135,136,137,138]

### 2.3. Bioinformatics-Based Approaches for Natural Products Discovery

Multiple genome-mining tools have been developed for the identification of secondary metabolite biosynthetic gene clusters, the most remarkable of which are ClustSCAN [139], NP searcher [140], GNP/PRISM [141] and antiSMASH [142]. Currently, antiSMASH is considered to be the most widely used tool for genome mining. It allows the prediction of a broad spectrum of secondary metabolite biosynthetic gene clusters [47,143].

Information about the composition of secondary metabolite BGCs gained with genome mining is crucial for the discovery of new secondary metabolites. It is a resource for synthetic biology-based rational design of BGCs and yields improvement strategies for target compounds. For example, polyketides and non-ribosomal peptides can be redesigned due to synthesis by connected modular enzymes that are able to recognize module-specific molecules [144].

### 2.4. Assembly Strategies for Generation of Constructs for Genetic Engineering of Streptomyces

To overcome challenges in cloning of actinobacterial sequences, new strategies to assemble constructs for genetic engineering of Actinobacteria were introduced, such as Gibson Assembly, BioBricks, iCatch, DiPaC and AGOS (Table 2) [50,56].

**Figure 4:**
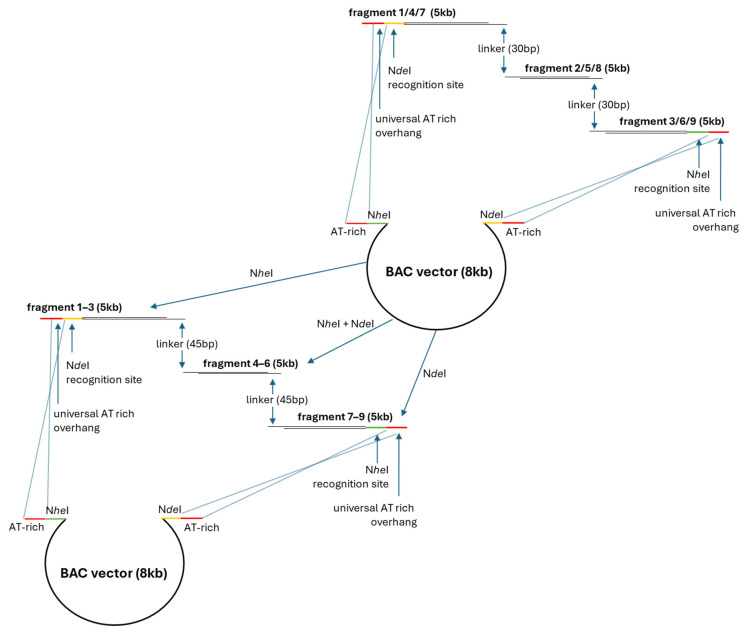
The modified Gibson assembly method for *Streptomyces*. Initially, three DNA fragments are assembled using a BAC vector. In the first step, the complementary overlaps between DNA inserts are 30 bp. Subsequently, the left, middle, and right assembled products from the first step are digested by *Nhe*I, *Nhe*I/*Nde*I, and *Nde*I, respectively. In the second step, the overlaps between DNA inserts are 45 bp.

Gibson Assembly [145] has become a common method for the genetic engineering of *Streptomyces*. This tool allows the assembly of multiple DNA fragments of up to 900 kb in size, which are cloned into a linearized vector requiring overlapping DNA sequences. However, the classical Gibson reaction has not been optimized for the cloning of large DNA fragments with high GC content, such as BGCs from *Streptomyces*. Using the original method, the self-ligation rate may reach 80% [146]. Adjustments of this method using terminal overhangs with high AT content and *Nde*I and *Nhe*I sites facilitated the assembly of large DNA molecules and decreased significantly the self-ligation rates (Figure 4) [146,147]. The applicability of the modified Gibson assembly protocol has been demonstrated for the assembly of the pristinamycin II (PII) cluster from *Streptomyces pristinaespiralis* HCCB10218 [146].

BioBricks is the technical standard for the physical composition of genetic parts, which should conform to the BioBrick assembly standard and are available from the Registry of Standard Biological Parts [148]. The toolkit consists of expression vectors, promoters, engineered *Streptomyces* hosts, etc. The BioBricks concept relies on the creation of DNA fragments flanked by the X*ba*I and S*pe*I restriction sites. This allows the generation of compatible cohesive ends and easy assembly of any desired DNA sequence [149].

A genetic engineering approach based on the BioBrick standard is the iCatch [150]. It is an upgrade of the iBrick principle [151]. This tool was designed to overcome the limitations of iBrick, which features difficulties in capturing large BGCs from *Actinomyces*. iCatch provides a possibility of capturing large actinobacterial gene clusters [150].

Other possibilities to assemble a complete *Streptomyces* BGC are Direct Pathway cloning (DiPaC) [152], sequence- and ligation-independent cloning (SLIC) [153] and the artificial gene operon assembly system (AGOS) [154]. DiPaC is conducted by covering full BGCs with long-amplicon PCR. Subsequent HiFi-based DNA assembly (e.g., Gibson assembly) is possible by the introduction of homologous nucleotide overhangs. DiPaC is suitable for cloning short to midsized BGCs in strains with high-GC content (Table 2) [152]. Using the SLIC method, the terminal homologous sequences of fragments anneal in vitro, whereas stitching of the gaps happens in vivo in the *E. coli* host [155]. AGOS is suitable for the reconstruction and assembly of gene operons, providing a set of entry plasmids designed for artificial gene operon manufacturing. Application of this strategy has been reported for the disassembly of large BGCs of up to 20 kb in size [154,156,157].

### 2.5. Genetic Approaches for Streptomyces Engineering

#### 2.5.1. Transposon- and Homologous Recombination-Based Systems for Actinomycetes Engineering—I-SceI Meganuclease-Promoted Recombination System

Such approaches as homologous recombination [20,158,159,160,161,162], transposons [163,164,165] and site-specific recombination [166,167,168] belong to the most extensively used tools for actinobacterial engineering (Table 2). However, the engineering of other Actinomycetales, such as *Planobispora* sp., *Kibdelosporangium* sp. and *Amycolatopsis* sp., remains challenging [48,163].

For systematic studies of microorganisms, transposon-based genome mutagenesis is a widely applied technique [169,170], but Tn*5* and IS*493*-derived transposons are not optimal for *Streptomyces* engineering [171]. To overcome this challenge, a codon-optimized, hyperactive Tn*5*-based transposition system random mutagenesis in *Streptomyces* was introduced, which was utilized to investigate the regulation of prodiginine in *S. coelicolor* [166].

In order to achieve deletions of large genome fragments in *Streptomyces*, the I-*Sce*I endonuclease [80] and the Cre recombinase [167,168] have been extensively used. I-*Sce*I meganuclease can recognize an 18-bp unique sequence (TAGGGATAACAGGGTAAT) and cause DNA double-strand breaks (DSBs) that promote double-crossover recombination events. This tool has been successfully validated in *S. coelicolor;* two BGCs that biosynthesize actinorhodin and undecylprodigiosin were successfully deleted by this technology [80].

#### 2.5.2. Cre/loxP- and Flp/FRT-Mediated Recombination

Another tool for advanced *Streptomyces* engineering is the Cre/loxP system from the P1 phage and the Flp/FRT system from yeast. The Cre and Flp proteins are bidirectional tyrosine recombinases that catalyze reciprocal SSR of DNA at 34-bp target sites (loxP and FRT, respectively). It results in either excision or inversion depending on whether the target sites are located as direct or inverted repeats. Both recombinases are context independent since they do not require any cofactors or accessory proteins from the host (Table 2) [166,167,168]. Both Flp and Cre recombinases have been successfully expressed in Actinobacteria. Successful expression of the Cre recombinase in *Streptomyces coelicolor* A3(2) was reported. Successful expression of two synthetic genes encoding the Cre and Flp recombinases were reported subsequently as well to delete resistance markers in members of the *Streptomyces* and *Saccharothrix* genera. The native Flp-encoding gene was also expressed in Streptomyces [167]. Recently, the Cre/loxP system was utilized for the excision of a large genomic region—1.3-Mb and 0.7-Mb were deleted in the natamycin producer strain *Streptomyces chattanoogensis* L10 [81,172].

#### 2.5.3. PCR-Targeted Gene Replacement

To overcome the challenges of classical strain engineering in *Streptomyces*, a PCR targeting-based gene disruption approach was introduced. It permits deletions of entire gene clusters and allows rapid generation of nonpolar, in-frame deletions, avoiding polar effects on downstream genes. This procedure is based on the discovery that allelic exchanges in the *E. coli* chromosome can be induced by recombination with a PCR-amplified selectable marker, which is flanked at both ends by extensions of a few tens nucleotides that are homologous to the desired region on the chromosome [50].

For this, the Redα (exo), Redβ (bet) and Redγ (gam) proteins of the phage λ should be present in the targeted strain. λ-Red can be used to promote recombination in *E. coli* between a PCR-amplified antibiotic resistance cassette selectable in *E. coli* and *Streptomyces*, and *S. coelicolor* DNA on a cosmid from the set used to sequence the *S. coelicolor* genome. An origin of transfer (oriT; RK2) included in the disruption cassette allows the transfer of the PCR-targeted cosmid into *S. coelicolor* by conjugation, readily yielding exconjugants with the desired gene replacement. This strategy also allows the elimination of the disruption cassette by FLP-recombinase-mediated site-specific recombination and was shown to be successfully applicable across *Streptomyces* strains (Table 2) [78].

#### 2.5.4. CRISPR/Cas9-Based Editing Tools in Streptomyces

**Figure 5:**
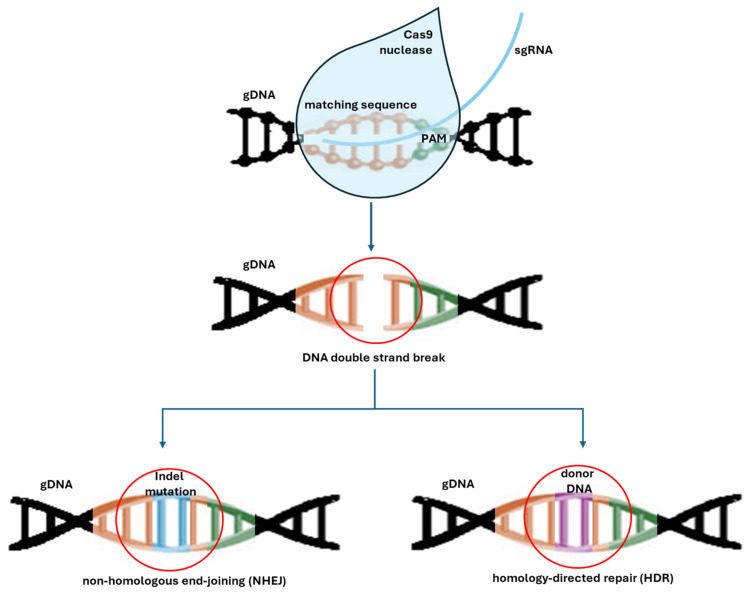
Schematic representation of the Cas-nuclease mediated generation of DNA double-strand breaks with subsequent endogenous DNA repair machinery. The guide RNA (sgRNA) contains a spacer/matching sequence that directs the Cas endonuclease to the complementary sequence. When the help of the protospacer adjacent motif (PAM), the Cas protein induces double-strand DNA breaks (DSB) in the target genome. During the non-homologous end-joining (NHEJ) pathway, repair proteins introduce insertions or Indels (deletions) because of the lack of a homolog repair template. If a homolog DNA segment is present (donor DNA), proteins of the homology-directed repair (HDR) system facilitate genomic recombination, enabling precise gene modifications.

Since the identification of CRISPR (clustered regularly short palindromic repeats) [173] and defense functions against phages [174] in combination with the CRISPR-associated (Cas) genes [175], the CRISPR/Cas system has recently emerged and been introduced as a promising tool for genome engineering in multiple organisms [176,177,178], including *Streptomyces* [179]. The CRISPR/Cas system typically consists of two components: a guide RNA and a Cas protein with endonuclease activity. The guide RNA contains a spacer sequence that directs the Cas endonuclease to its complementary protospacer sequence. A scaffold facilitates this interaction [60]. The guided Cas protein induces double-strand DNA breaks (DSBs) in the target genome through two nuclease domains, HNH (complementary strand) and RuvC-like (non-complementary strand), when the correct protospacer adjacent motif (PAM) is present [60]. That is followed by repairing the DSB via native non-homologous end joining repair (NHEJ) or homology-directed repair (HDR) mechanisms. During DNA repair via the NHEJ pathway, small insertions and/or deletions may occur randomly at the DNA cleavage site (Figure 5) [60,179].

CRISPR/Cas systems can facilitate unmarked genome engineering with reduced time and effort. CRISPR/Cas system has become a dominant genome engineering tool nowadays, due to the possibility of avoiding the design of proteins for individual target sequences when using alternative similar strategies, like zinc finger nuclease or transcription activator-like effector nuclease [60]. Genes involved in the bacterial NHEJ pathway are absent in most *Streptomyces*. Thus, genome editing mediated by the HDR pathway is preferred for CRISPR/Cas-based genetic engineering [180]. Achieving scarless genome editing in *Streptomyces* was mostly dependent on homologous recombination double-crossover events that required time-consuming and labor-intensive screening steps [20]. Given the lethal DSB induced by the CRISPR/Cas system in bacteria unless repaired, the surviving cells after transformation of the CRISPR/Cas plasmid are more likely to be successfully engineered [179,180,181,182].

The first CRISPR/Cas system applied to *Streptomyces* was based on the class 2–type II CRISPR system using Cas9 nuclease from *Streptococcus pyogenes*. Cas9 requires 5′-NGG-3′ as the PAM, which is frequently found in the GC-rich genomes of *Streptomyces*. Using the CRISPR/Cas9 system, genomic deletions of up to 31.4 kbp have been performed in *S. lividans*, *S. albus* and *S. viridochromogenes* [179]. Additional CRISPR/Cas9 systems were explored subsequently leading to enhanced editing efficiency that facilitates rapid gene disruption [181,182,183]. Although most CRISPR/Cas9 systems rely on a temperature-sensitive origin to cure the plasmid, an alternative counterselection method for screening plasmid-cured strains has been described, based on a mutant cytosine deaminase that can convert 5-fluorocytosine into the highly toxic 5-fluorouracil. It was integrated into the CRISPR/Cas9 plasmid, and treatment with 5-fluorocytosine effectively eliminated the strains retaining the plasmid [184].

The CRISPR/Cas system for *Streptomyces* recently evolved by exploiting different types of CRISPR/Cas systems and expanding its applicable *Streptomyces* hosts, including *S. rimosus*, *S. ambofaciens* and *S. roseosporus* [60,185,186,187,188]. The applicability of a specific type of CRISPR system may differ from species to species [187,189]. The class 2–type V CRISPR/Cas system, exploiting Cas12a from *Francisella novicida*, expanded the number of editable DNA sequences with 5′-TTV-3′ PAM [120]. Although the 5′-NGG-3′ PAM of Cas9 may provide a wide range of potential editing targets, the AT-rich PAM of Cas12a provided advantages for targeting the GC-rich genome of *Streptomyces*. Cas12a is also more suited for multiplexing compared to Cas9 because it enables the maturation of CRISPR RNA (crRNA) (Table 2) [190,191].

Both CRISPR/Cas9 and CRISPR/Cas12a are convenient to use because only a single Cas protein is required to induce DSB in the genome [192]. The introduced foreign CRISPR/Cas system may crosstalk with the native CRISPR/Cas system encoded within the genome, resulting in reduced engineering efficiency and defects in the host [193]. The class 1 type I CRISPR/Cas system requiring a Cas3 nuclease and a ribonucleoprotein complex comprising Cas5, Cas7 and Cas8 proteins, as well as the crRNA, is the most widespread CRISPR/Cas system in *Streptomyces* because class 1 type I CRISPR/Cas was shown to appear advantageous for a broad range of *Streptomyces* species (Table 2) [187].

The CRISPR/Cas approach has been recently applied to identify BGCs of multiple secondary metabolites, such as sceliphrolactam, valinomycin and griseusin. This was achieved by introducing a stop codon in the synthesis genes or by deleting biosynthetic genes in respective BGCs [194]. BGC disruption enabled functional screening of BGCs. For example, in *S. globisporus* SP6C4, the biosynthetic genes of 15 BGCs were deleted using the CRISPR/Cas system, and the BGC-disrupted strains were tested for antifungal and antibacterial activities, revealing three bioactive lantipeptide- or lassopeptide-type secondary metabolites [194].

#### 2.5.5. CRISPR/Cas9 TAR Cloning Approach

In order to clone large DNA fragments of up to 250 kb, the TAR cloning (transformation-associated recombination) approach has been introduced [195]. This method allows for bypassing the construction of random clone libraries. TAR cloning is based on homologous recombination of a specific genome target and on a linear vector containing anchor-like, specific terminal targeting sequences, which are homologs to the sequences of interest. First, the vector and genomic DNA are introduced into yeast. Afterward, homologous recombination of the flanking regions of the target sequences and the vector anchors takes place, resulting in Yeast Artificial Chromosomes (YACs) with the target sequence.

**Figure 6:**
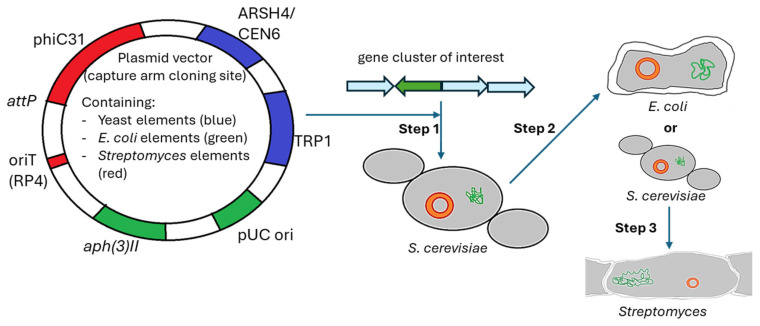
Design and strategy of TAR-based cloning and expression. Plasmid vectors should contain genetic elements for *Saccharomyces* (blue), *Escherichia coli* (green) and *Streptomyces* (red). In a three-step process, plasmid vectors are first introduced into yeast, where recombination events with the target BGC result in generation of Yeast Artificial Chromosomes (YACs). Afterwards, the genetic construct is transferred into the *Streptomyces* host through an intermediate yeast or *E. coli* host.

The combination of CRISPR/Cas with the TAR cloning optimized the specificity of this approach by 30%—almost any sequence of interest can be cloned into a YAC vector, without the need for specific and rare restriction enzymes that cut near the target sequence. This method was applied for the cloning of the pristinamycin BGC from *S. pristinaespiralis* and the prodigiosin BGC from *S. coelicolor* (Figure 6) [195].

BGCs can also be cloned using the Cas9-assisted targeting of chromosomal segments (CATCHs) method that uses a combination of Gibson assembly for cloning genomic DNA fragments into linearized DNA vectors and in vitro Cas9 digestion of genomic DNA. A limitation of this method is the cloning of large actinobacterial BGCs (>50 kb) from high GC-content organisms (Table 2) [196].

**Table 2:** A combined list of synthetic biology technologies for genetic manipulation of *Streptomyces*.

Technology	Category	Feature	References
Target BGC acquisition	Genetic manipulation strategy	Transfer into a target host using a genomic library of cosmids, fosmids, BAC, PAC, BioBrick	[75,82,92,148,149]
Ligation and BGC assembly to the vector	Genetic manipulation strategy	Sticky/blunt end ligation, Gibson cloning, recombination in different hosts, etc.	[50,56,61,145,146]
Transfer of the BGC-encoded vector to the heterologous host for expression	Genetic manipulation strategy	Biparental conjugation, protoplast transformation	[56]
Target secondary metabolite production by expression of the BGC vector	Genetic manipulation strategy	Expression of integrative (e.g., pSET152, pIJ8600, pOJ436, pOJ444) or replicative (e.g., pUWL218, pUWL219, pUWL-SK, pUWL-KS) vectors	[75,82,92]
iCatch	Assembly strategy	Facilitate “catching” of large DNA regions like actinobacterial BGCs	[150]
DiPAC	Assembly strategy	Assembly of complete biosynthetic pathways by covering full BGCs with long-amplicon PCR	[152]
AGOS	Assembly strategy	Reconstruction and assembly of gene operons	[153]
PCR-targeting system	Genetic manipulation strategy	Nonpolar/in-frame deletion of genes/gene clusters in *Streptomyces*	[78]
Cre-loxP/Flp-FRP recombination systems	Genetic manipulation strategy	Knock out large DNA fragments in *Streptomyces*	[79,166,167,172]
I-SceI promoted recombination system	Genetic manipulation strategy	DNA double-strand breaks (DSBs), which promote double-crossover recombination events	[80]
SpCas9-based genome editing	Genetic manipulation strategy	Transcribed synthetic guide RNA to direct Cas proteins to any site on the genome. Editing plasmids: pCRISPomyces-1/2, pKCas9dO, pCRISPR-Cas9-ScaligD, and pWHU2653	[180,181,182]
CRISPRi-mediated gene repression for single cells	Genetic manipulation strategy	Gene repression tool based on dCas9 or ddCpf1 and the base editors (BEs) for targeted base mutagenesis based on dCas9 or Cas9n	[181]
FnCpf1-based genome editing and CRISPRi	Genetic manipulation strategy	Editing plasmids: pKCCpf1, pKCCpf1-MsmE, and pSETddCpf1, etc.	[188]
CRISPR/Cas-based base editing tools	Genetic manipulation strategy	Editing plasmids: pCRISPR-cBEST/-aBEST, and pKC-dCas9-CDA-ULstr, etc.	[60,180,181]
Alternative CRISPR/Cas-based genome editing	Genetic manipulation strategy	Editing plasmids: pCRISPomyces-FnCpf1, pCRISPomyces-Sth1Cas9, and pCRISPomyces-SaCas9, etc.	[186,187,188,189]
TAR-cloning	Genetic manipulation strategy	Isolation of large chromosomal regions without the constructing a random clone library	[194]
CATCH	Genetic manipulation strategy	A combination of in vitro Cas9 digestion of genomic DNA in agarose gel-plugs and isothermal Gibson assembly for cloning genomic DNA fragments into linearized DNA vectors	[196]

## 3. Conclusions and Future Perspectives

The complex biosynthesis of secondary metabolites in *Streptomyces* wild-type producers naturally results in rather low titers. Biosynthetic steps in primary and secondary metabolism, as well as their regulation, represent targets for engineering. The dependence of secondary metabolism in *Streptomyces* spp. on precursor supply coming from primary metabolism is crucial and can be optimized for biotechnological needs. However, genetic engineering of *Streptomyces* has historically been challenging. New tools proposed recently accelerated genetic manipulations of *Streptomyces*, introducing a growing number of new approaches for BGC and metabolic engineering. The traditional method for the generation of deletion mutants is time-consuming. Novel approaches, such as the CRISPR/Cas system, TAR-cloning, PCR-targeting or Cre/loxP recombination were adapted to *Streptomyces* for fast genome editing. For instance, several optimized CRISPR/Cas-based technologies were recently reported to improve the turnaround time for the generation of deletion mutants even further. Implementation of new systems resulted in the efficient genome engineering of a broad range of *Streptomyces* strains and some other actinomycetes. Newly developed tools provide new opportunities for natural product discovery and metabolic engineering of *Streptomyces*. It is likely that this progress will continue, and additional cell factories can be developed for the development and manufacturing of various valuable products.

## Data Availability

No new data were created or analyzed in this study.
